# Dynamic Changes in Fluid Temperatures during Laser Irradiation Using Various Laser Modes: A Thermography-Based In Vitro Phantom Study

**DOI:** 10.3390/jcm12041409

**Published:** 2023-02-10

**Authors:** Shimpei Yamashita, Takaaki Inoue, Satoshi Imai, Yohei Maruyama, Yuya Iwahashi, Ryusuke Deguchi, Yasuo Kohjimoto, Masato Fujisawa, Isao Hara

**Affiliations:** 1 Department of Urology, Wakayama Medical University, 811-1 Kimiidera, Wakayama City 641-0012, Japan; 2 Department of Urology, Hara Genitourinary Hospital, 5-7-17 Kitanagasadori, Chuo-ku, Kobe City 650-0012, Japan; 3 Department of Urology, Kobe University, 7-5-1 Kusunoki-cho, Chuo-ku, Kobe City 650-0017, Japan; 4 Department of Urology, Konan Medical Center, 1-5-16 Kamokogahara, Higashinada-ku, Kobe City 658-0064, Japan

**Keywords:** ureteroscopy, retrograde intrarenal surgery, laser lithotripsy, thermal injury, thermography, Moses technology

## Abstract

The differences in dynamic thermal changes during laser lithotripsy between various laser pulse modes are unclear. We used thermography to evaluate the temporal changes in high-temperature areas during laser activation in order to compare different laser pulse modes. An unroofed artificial kidney model was used for the experiments. The laser fired for 60 s with a laser setting of 0.4 J/60 Hz in the following four different laser pulse modes without saline irrigation: short pulse mode (SPM), long pulse mode (LPM), virtual basket mode (VBM) and Moses mode (MM). Using the first 30 s of moving images, we compared the ratio of a high-temperature area of >43 °C to the total area every 5 seconds. The dynamic changes in fluid temperatures were shown to be different between the laser pulse modes. The extent of the high-temperature areas during the laser activation was large in the LPM and MM compared with the SPM and VBM. While the high-temperature areas expanded in an anterior direction in the early laser irradiation period using the LPM, they spread in a posterior direction in the early laser activation period using the MM. Although only the temperature profile in one specific plane was investigated, these results are considered useful for preventing thermal injuries during retrograde intrarenal surgeries.

## 1. Introduction

With recent advances in surgical technology in the endourological field, retrograde intrarenal surgeries (RIRSs) using a flexible ureteroscope (fURS) and a holmium: yttrium–aluminum–garnet (Ho:YAG) laser have become a standard surgical method for renal stones [1]. Although RIRSs using the Ho:YAG laser are relatively safe and effective, considering that urolithiasis is a benign disease, the prevention of severe perioperative complications is as important as the achievement of a stone-free status.

Recently, thermal injuries, which are indirect injuries to the urothelial mucosa caused by elevations in irrigation temperatures due to laser irradiation, have gained attention in relation to high-power laser systems (100–120 W) because of the risk of developing ureteral strictures or reducing renal function [2]. To prevent perioperative complications, the excessive elevation in irrigation temperatures during laser irradiation should, therefore, be avoided. Various factors could influence the irrigation temperatures, including laser energies, laser irradiation times and irrigation conditions [2,3]. In addition, the laser pulse mode has also been reported to affect irrigation temperatures [4]. The long pulse mode (LPM) could cause a higher rise in irrigation temperatures than the short pulse mode (SPM). A pulse modulation mode based on Moses technology, known as the Moses mode (MM), may reduce the risk of temperature elevation in irrigation fluids compared with the LPM. Moreover, our thermography-based in vitro phantom study suggested that although the virtual basket mode (VBM) is also a pulse modulation mode that uses Moses technology, there might be a more rapid rise in temperature in the MM than in the VBM [5]. However, the difference in impact on the irrigation temperatures between these two modes has not been sufficiently compared. In addition, to the best of our knowledge, there is no published data on the dynamic thermal changes during laser irradiation using various laser pulse modes.

This study uses thermography and compares various laser pulse modes, such as the MM and VBM, to evaluate the dynamic thermal changes in irrigation temperatures within an area and the spread of high temperatures during laser irradiation.

## 2. Materials and Methods

**Figure 1:**
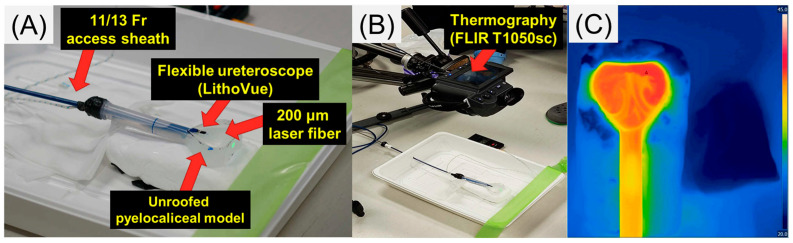
(**A**) Experimental setup; (**B**) thermography setup; (**C**) moving image of the irrigation temperatures.

The experimental setup is shown in Figure 1A,B. A 50 mm silicon flask (outer diameter: 50.5 mm; height: 150 mm, VITLAB, Grossostheim, Germany) was used to create an unroofed artificial kidney model. An 11/13-Fr ureteral access sheath (Navigator, Boston Scientific Co., Marlborough, MA, USA) was connected to it, and a disposable ureteroscope (LithoVue, Boston Scientific Co., Marlborough, MA, USA) with a 200 μm laser fiber was inserted into the model through the sheath. The model and ureteral access sheath were filled with 37 °C saline. The distance between the saline surface and the tip of the laser fiber was measured using a ruler and set to 5 mm. The FLIR T1050sc (Teledyne FLIR LLC, Wilsonville, OR, USA), which is a thermographic camera, was fixed at 40 cm from the water surface. It captured moving images of the dynamic changes in the irrigation temperatures (Figure 1C).

In this experiment, the Cyber Ho 100W (Quanta System, Milan, Italy) and Pulse 120H (Lumenis, Yokneam, Israel) high-power Ho:YAG laser systems were used, with the laser set at 0.4 J/60 Hz. The laser fired for 60 s using the following four different laser pulse modes: SPM, LPM, VBM (using Cyber Ho 100W) and MM (contact, using Pulse 120H) without saline irrigation. Three runs were tested for each laser pulse mode.

Based on a previous study that suggested that thermal injuries occurred at temperatures of >43 °C, the threshold for thermal injuries was defined as 43 °C in this study [4]. Using the first 30 s of moving images from the start of the laser irradiation, which were provided by thermography, we recorded the maximum temperatures of the water surface and the ratio of the high-temperature area above 43 °C to the total area every 5 seconds in each session. After processing the images to convert the high-temperature areas into red-colored areas, the ratios of the high-temperature areas above the threshold were calculated using open-source software (https://imagej.net/ij/, accessed on 25 December 2022). The ratios were also analyzed separately for the anterior and posterior areas of the laser tip. We compared these outcomes between the different laser pulse modes using ANOVA tests and Turkey-Kramer HSD tests. The results are shown as a mean ± standard error of the mean. All statistical analyses were performed using JMP Pro 14 (SAS Institute Inc., Cary, NC, USA). For all statistical tests, the significance threshold was *p* < 0.05.

## 3. Results

### 3.1. Comparison of the Maximum Temperatures of the Water Surface

**Figure 2:**
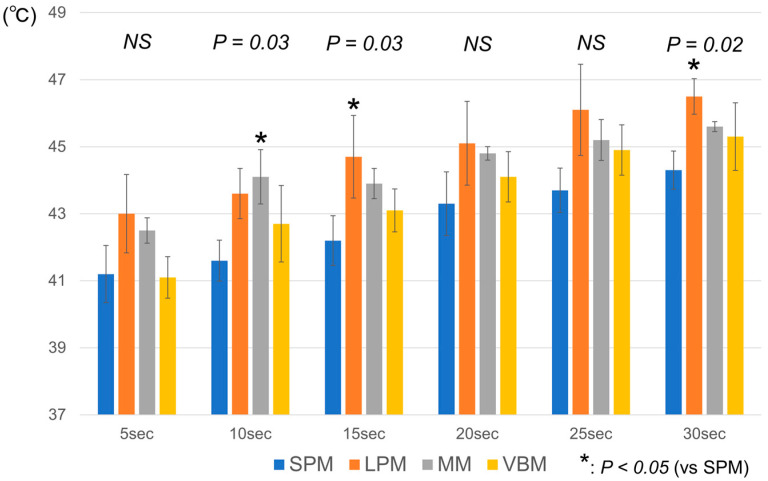
Maximum irrigation temperatures of different laser pulse modes. SPM: short pulse mode; LPM: long pulse mode; MM: Moses mode; VBM: virtual basket mode; NS: not significant.

Comparisons of the maximum temperatures of the water surface every 5 seconds during the 30-s period are shown in Figure 2. The maximum temperatures were significantly different between the laser pulse types at 10, 15 and 30 s (*p* = 0.03, *p* = 0.03 and *p* = 0.02, respectively). At 10 s, the maximum temperature in the MM (44.1 ± 0.8 °C) was significantly higher than that in the SPM (41.6 ± 0.6 °C) (*p* = 0.03). At 15 and 30 s, the maximum temperatures in the LPM (44.7 ± 1.2 °C and 46.5 ± 0.5 °C, respectively) were significantly higher than those in the SPM (42.2 ± 0.7 °C and 44.3 ± 0.6 °C, respectively) (*p* = 0.02 and *p* = 0.01, respectively).

### 3.2. Comparison of the Ratios of High-Temperature Areas above the Threshold

**Figure 3:**
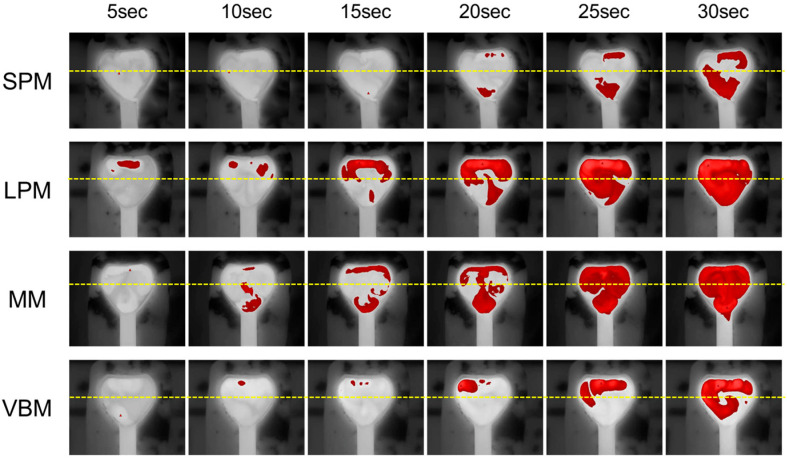
Examples of high-temperature area distributions. The red areas show high temperatures above the threshold. The yellow lines indicate the boundary between the region anterior and posterior of the laser tip. SPM: short pulse mode; LPM: long pulse mode; MM: Moses mode; VBM: virtual basket mode.

Figure 3 shows examples of high-temperature area distributions. The sizes and distributions of the high-temperature areas seemed to differ between the laser pulse modes.

**Figure 4:**
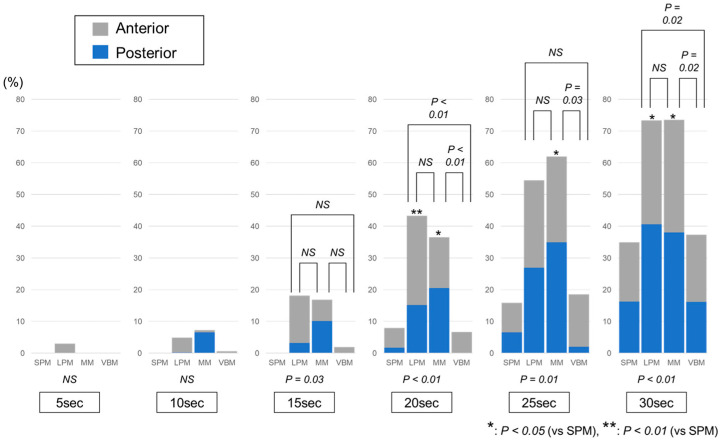
Comparison of the ratios of the total high-temperature areas. The gray and blue bars show the ratios of the high-temperature areas anterior and posterior to the laser tip, respectively. SPM: short pulse mode; LPM: long pulse mode; MM: Moses mode; VBM: virtual basket mode.

Comparisons of the ratios of the total high-temperature areas among the laser pulse modes are shown in Figure 4. The ratios of the high-temperature areas were significantly different between the laser pulse modes at 15, 20, 25 and 30 s (*p* = 0.03, *p* < 0.01, *p* = 0.01 and *p* < 0.01, respectively). At 20 s, the ratios in the LPM (43.3 ± 8.0%) and MM (36.6 ± 9.7%) were significantly higher than those in the SPM (8.0 ± 9.8%) (*p* < 0.01 and *p* = 0.01, respectively) and VBM (6.7 ± 4.3%) (*p* < 0.01 and *p* < 0.01, respectively). At 25 s, the ratio in the MM (62.0 ± 23.2%) was significantly higher than the ratios in the SPM (15.9 ± 15.4%) and VBM (18.5 ± 9.4%) (*p* = 0.02 and *p* = 0.03, respectively). At 30 s, the ratios in the LPM (73.4 ± 7.9%) and MM (73.6 ± 9.2%) were significantly higher than those in the SPM (35.0 ± 13.9%) (*p* = 0.01 and *p* = 0.01, respectively) and VBM (37.3 ± 15.8%) (*p* = 0.02 and *p* = 0.02, respectively).

**Figure 5:**
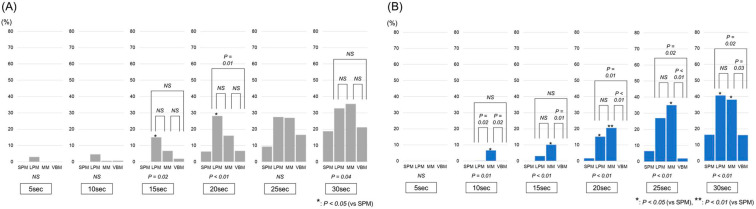
Comparison of the ratios of the high-temperature areas (**A**) anterior and (**B**) posterior to the laser tip. SPM: short pulse mode; LPM: long pulse mode; MM: Moses mode; VBM: virtual basket mode.

Comparisons of the ratios of the high-temperature areas anterior to the tip of the laser fiber are shown in Figure 5A. The ratios of the high-temperature areas to the front of the laser tip were significantly different between the laser pulse modes at 15, 20 and 30 s (*p* = 0.02, *p* < 0.01 and *p* = 0.04, respectively). At 15 s, the ratio in the LPM (15.0 ± 7.9%) was significantly higher than that in the SPM (0.0 ± 0.0%) (*p* = 0.02). At 20 s, the ratio in the LPM (28.1 ± 0.7%) was significantly higher than the ratios in the SPM (6.3 ± 10.0%) and VBM (6.7 ± 4.3%) (*p* = 0.01 and *p* = 0.01, respectively). Conversely, as shown in Figure 5B, the ratios of the high-temperature areas behind the laser tip were significantly different between the laser pulse modes at 10, 15, 20, 25 and 30 s (*p* = 0.01, *p* < 0.01, *p* < 0.01, *p* < 0.01 and *p* < 0.01, respectively). At 10 s, the ratio in the MM (6.6 ± 4.5%) was significantly higher than the ratios in the SPM (0.0 ± 0.0%), LPM (0.3 ± 0.4%) and VBM (0.0 ± 0.0%) (*p* = 0.02, *p* = 0.03 and *p* = 0.02, respectively). At 15 s, the ratio in the MM (10.2 ± 3.6%) was significantly higher than the ratios in the SPM (0.0 ± 0.0%) and VBM (0.0 ± 0.0%) (*p* = 0.01 and *p* = 0.01, respectively). At 20 s, the ratios in the LPM (15.2 ± 7.6%) and MM (20.6 ± 4.3%) were significantly higher than those in the SPM (1.7 ± 2.1%) (*p* = 0.02 and *p* < 0.01, respectively) and VBM (0.0 ± 0.0%) (*p* = 0.01 and *p* < 0.01, respectively). At 25 s, the ratio in the MM (35.0 ± 11.5%) was significantly higher than the ratios in the SPM (6.6 ± 5.9%) and VBM (2.0 ± 2.6%) (*p* = 0.01 and *p* < 0.01, respectively). Moreover, the ratio in the LPM (27.0 ± 9.3%) was also significantly higher than that in the VBM (*p* = 0.02). At 30 s, the ratios in the LPM (40.7 ± 2.9%) and MM (38.1 ± 7.4%) were significantly higher than those in the SPM (16.3 ± 12.1%) (*p* = 0.02 and *p* = 0.03, respectively) and VBM (16.2 ± 6.2%) (*p* = 0.02 and *p* = 0.03, respectively).

## 4. Discussion

We used thermography to investigate the dynamic thermal changes in irrigation temperatures, including the area and the spread of the high temperatures during laser irradiation, and compared them between various laser pulse modes, such as the MM and VBM. The laser pulse modes not only affected the maximum temperatures of the irrigation fluid but also the area and the spread of the high temperatures during RIRS.

Laser lithotripsy using the Ho: YAG laser has become a common surgical procedure for urinary stone disease since its introduction in the 1990s [6]. Although the exact mechanisms of stone ablation by the laser are still poorly understood, two possible mechanisms, namely the photothermal effect and the photomechanical effect, have been reported [7,8]. The photothermal effect is associated with electromagnetic radiation, where the chemical decomposition of the stone components is heated by laser activation or boiling and vaporizing water. The photomechanical effect is associated with the vaporization of water inside the stone pores, followed by the stones bursting from the inside. However, in these laser lithotripsy processes, the fluid around the stones in the collecting system could have a significant rise in temperature, which could cause thermal injuries to the surrounding urothelial mucosa. Both the peak temperature and the exposure time reportedly contribute to the thermal cytotoxic effect [9]. The optimal cutoff temperature was reported to be 43 °C, which was also used as the threshold value in our study.

The irrigation temperatures in laser lithotripsy are known to depend on various factors, including irrigation conditions, laser power settings and continuous irradiation times. In previous studies, when a sufficient irrigation flow was available, the safety threshold of the irrigation temperature was not reached, even during high-power laser lithotripsy [3,10]. However, depending on the stone location, the condition of the urinary tract and the presence or size of a ureteral access sheath, a sufficient irrigation condition is not often available. To prevent thermal injuries, surgeons should perform safe laser lithotripsy with the appropriate laser settings. High-power laser settings and longer continuous laser activation times could cause a greater increase in the irrigation temperatures; thus, low-power laser settings and intermittent laser irradiation might be required under insufficient irrigation conditions [2,10].

Recently, laser pulse modes have also been reported to affect the temperature rise of irrigation fluids. The recent advancements in laser technology enable a choice of various laser pulse modes. The use of the LPM could result in a greater rise in irrigation temperatures compared with the use of the SPM [4]. In addition, the influence of pulse modulation modes, such as MM and VBM, on irrigation temperatures has also been investigated. Both the MM and VBM are two-staged laser shot modes based on Moses technology and available by using Pulse 120H and Cyber Ho 100W, respectively [11,12]. In these modes, the second laser pulse penetrates the bubble of the first laser pulse. Winship et al. reported that the MM produces significantly lower temperatures compared with the LPM [4]. In addition, the irrigation temperature in the MM could rise more rapidly than that in the VBM [5]. However, the influence of these laser pulse modes on dynamic changes in the area or the spread of high temperatures remains unclear. To the best of our knowledge, this is the first study to use thermography to evaluate dynamic irrigation temperatures by comparing four different laser pulse modes.

Our results led to two important clinical observations. First, the extent of a high irrigation temperature area could depend on the laser pulse modes. As shown in Figure 4, the ratios of the high-temperature areas were significantly different between the laser pulse modes at 15 s or later. The ratios of the high-temperature areas in the LPM and MM tended to be higher than those in the SPM and VBM. These results are understandable considering that the time to reach a threshold in the LPM and MM is significantly shorter than that in the SPM and VBM [5]. However, the exact etiology of the differences in heat production between different laser pulse modes remains unclear. A review of the laser fragmentation process showed that long pulse durations could result in thermal injuries to the surrounding soft tissues [13]. Nonetheless, the reason for the differences in the impact on thermal injury between standard pulse modes (SPM and LPM) and pulse modulation modes (MM and VBM) has not yet been completely elucidated. Moreover, the reason for the differences in the irrigation temperature rise between the two types of pulse modulation modes, MM and VBM, also remains unclear. Second, the spreading direction of the high-temperature areas was also different between the laser pulse modes. As described above, the ratios of the high-temperature areas were high in the LPM and MM. However, as shown in Figure 5, while the high-temperature area in the LPM tended to spread forward of the tip of the laser fiber in the early phase, it tended to spread backwards in the MM. In addition, although both the MM and VBM are pulse modulation modes based on Moses technology, the ratio of the high-temperature area backward of the tip of the laser fiber in the VBM was relatively low. These differences may be due to differences in water flow generated by the laser shots among the laser pulse modes, but there is little evidence about this point.

Our results require verification and elucidation of the differences in impact on the dynamic changes in irrigation temperature between the laser pulse modes. Nonetheless, these observations are perhaps informative in the prevention of thermal injuries during RIRS. Continuous laser irradiation should be avoided, especially when performing laser lithotripsy using the LPM or MM, to prevent thermal injuries to the surrounding urothelial mucosa because the high temperature areas can spread widely. Moreover, when using the MM, attention should be paid not only to the urothelial mucosa appearing in the endoscopic images but also to the mucosa posterior to the tip of the laser fiber. Careful consideration of the most appropriate laser pulse mode and laser irradiation method is required on a case-by-case basis.

Our study has several limitations. The artificial kidney model was unroofed to enable evaluation using thermography, so there might be some differences in the irrigation flow during the laser irradiation between our experiment and actual RIRS procedures. Second, the dynamic changes in irrigation temperatures in deep areas could not be investigated because thermography can only evaluate water surface temperatures. Third, only one laser setting, 0.4 J/60 Hz, was used in the current study. The dusting technique, which has become a popular technique in RIRS, uses high-power laser settings and requires a low pulse energy of 0.2–0.5 J and a high frequency of 2–80 Hz [14,15]. The laser settings available for both laser systems were limited; thus, this laser setting was used. Although our laser settings seem reasonable, different laser settings should also be evaluated in the future. In addition, we were unable to investigate the exact pulse duration and the actual pulse energy. The actual pulse energy can vary slightly between laser devices, and this difference might have an influence on the heat production. Finally, we only performed three runs with each set parameter. Further studies are required to examine the reproducibility of our results and verify our findings.

## 5. Conclusions

Our thermography-based in vitro phantom study demonstrated dynamic changes in the irrigation temperatures between different laser pulse modes. The extent of the high-temperature areas during the laser activation was large in the LPM and MM compared with the SPM and VBM. While the high-temperature areas expanded in an anterior direction in the early laser irradiation period using the LPM, the areas spread in a posterior direction in the early laser activation period using the MM. These results are considered useful for the prevention of thermal injuries during RIRS.

## Data Availability

Not applicable.
